# Waterborne Exposure of Paclobutrazol at Environmental Relevant Concentration Induce Locomotion Hyperactivity in Larvae and Anxiolytic Exploratory Behavior in Adult Zebrafish

**DOI:** 10.3390/ijerph17134632

**Published:** 2020-06-27

**Authors:** Akhlaq Hussain, Gilbert Audira, Petrus Siregar, Yi-Chen Lin, Omar Villalobos, Oliver Villaflores, Wen-Der Wang, Chung-Der Hsiao

**Affiliations:** 1 Department of Bioscience Technology, Chung Yuan Christian University, Chung-Li 32023, Taiwan; anjumarman390@gmail.com (A.H.); gilbertaudira@yahoo.com (G.A.); siregar.petrus27@gmail.com (P.S.); 2 Department of Chemistry, Chung Yuan Christian University, Chung-Li 32023, Taiwan; 3 Department of Bioagricultural Science, National Chiayi University, Chiayi 60004, Taiwan; cdhsiao@gmail.com; 4 Department of Pharmacy, Faculty of Pharmacy, University of Santo Tomas, Manila 1015, Philippines; oavillalobos@ust.edu.ph; 5 Department of Biochemistry, Faculty of Pharmacy, University of Santo Tomas, Manila 1015, Philippines; obvillaflores@ust.edu.ph; 6 Center for Nanotechnology, Chung Yuan Christian University, Chung-Li 32023, Taiwan

**Keywords:** Paclobutrazol, biosafety, antioxidant response, anxiolytic behavior

## Abstract

The available arable land is unable to fulfill the food production need of rapidly the exponentially growing human population in the world. Pesticides are one of those different measures taken to meet this demand. As a plant growth regulator to block gibberellin, paclobutrazol (PBZ) is used excessively throughout the world to promote early fruit setting, and to increase seed setting which might be harmful because PBZ is a very stable compound; therefore, it can bioaccumulate into the food chain of an ecosystem. In the present study, we discovered unexpected effects of PBZ on zebrafish larvae and adult behaviors by challenging them with low dose exposure. Zebrafish larvae aged 4 days post-fertilization (dpf) were exposed for 24 h at 10 µg/L (0.01 ppm) and 100 µg/L (0.1 ppm) of PBZ, respectively, and adults were incubated at 100 µg/L (0.1 ppm) and 1000 µg/L (1 ppm) concentrations of PBZ, respectively, for fourteen days. After incubation, the locomotor activity, burst, and rotation movement for the larvae; and multiple behavioral tests such as novel tank exploration, mirror biting, shoaling, predator avoidance, and social interaction for adult zebrafish were evaluated. Brain tissues of the adult fish were dissected and subjected to biochemical analyses of the antioxidant response, oxidative stress, superoxide dismutase (SOD), and neurotransmitter levels. Zebrafish larvae exposed to PBZ exhibited locomotion hyperactivity with a high burst movement and swimming pattern. In adult zebrafish, PBZ resulted in anxiolytic exploratory behavior, while no significant results were found in social interaction, shoal making, and predator avoidance behaviors. Interestingly, high dose PBZ exposure significantly compromised the innate aggressive behavior of the adult fish. Biochemical assays for oxidative stress, antioxidant response, and superoxide dismutase (SOD) showed significant reductions in their relative contents. In conclusion, for the first time, our behavior assays revealed that chronic PBZ exposure induced behavioral alterations in both larvae and the adult zebrafish. Because PBZ is a widely-used plant growth regulator, we suggest that it is necessary to conduct more thorough tests for its biosafety and bioaccumulation.

## 1. Introduction

Pesticides are considered as one of the major factors in the development of agricultural yield in the 20th century [1]. Organic pesticides and their active ingredients are being used widely in agriculture sector. The synthetic organic herbicides and pesticides play a major role in the agricultural development since 1940. More than 16,000 formulations of pesticides and 1055 different types of their active ingredients have been registered in 2014 [2,3]. With the minor advantages, pesticides and formulations have a worsening effect on our environment. Approximately three million individuals in developing countries are poisoned by pesticides and two million peoples died each year [4]. Due to their potential adverse effects on the environment and humans, the application of many pesticides and formulations have been banned. Most of the organochlorine and organophosphate compounds were banned. Compared to other pesticides, Triazole emerged as being widely used worldwide because it was considered environment-friendly [5]. Paclobutrazol (PBZ) herbicides are one of the Triazoles being mostly used nowadays. PBZ suppresses aerial growth by inhibiting the plant growth hormone gibberellin biosynthesis, which is helpful for increasing seed setting, and promote early fruit setting in plants. To date, 136 types of gibberellins have been identified, amongst which GA1, GA3, GA4, and GA7 are known to be bioactive [6]. In plants, the enzyme ent-kaurene oxidase that is majorly involved in gibberellin biosynthesis is blocked by PBZ. PBZ is used on flowers, fruits, vegetables, and other crops to enhance flowering and food setting. PBZ also plays an active role to safeguard crops from several environmental stresses including, heat radiation, chilling, and drought [7]. The method of application of PBZ includes soaking the seeds, foliar spray [8], method drenching over the stems/leaves, and exposing the powder to roots to be transferred in the plant through the xylem [9].

The level of PBZ contamination in the soil is estimated to be 150 mg/kg, and the concentration in the groundwater is estimated to be 4.2 µg/L (0.0042 ppm) and the surface water of the river is 119.6 ng/L (ppb) [10]. Chemically, PBZ is a very stable compound with a half-life of 182 days and 164 days in the soil and surface water, respectively. Therefore it can easily bioaccumulate in foods and alter the food chain of an ecosystem [11]. The effect of PBZ on the activities of antioxidant enzymes such as superoxide dismutase (SOD) and catalase (CAT) has been studied in zebrafish (*Danio rerio)* [10]. Impairment of embryonic development, disruption of spermatogenesis in zebrafish, and hepatic steatosis in Sea ruffe (*Sebastiscus marmoratus*) have been reported in previous studies [10,12]. Furthermore, PBZ exposure induced morphological malformation of the heart, eyes, and head in zebrafish embryos [13,14]. Far less is known, however, about the toxicity effects of PBZ exposure on the behaviors of larvae and adult zebrafish in terms of neurotransmitters and antioxidant enzymes activity on behavior endpoints. As the concentration of PBZ in river water was described 119.6 ng/L which is equal to 0.119 ppb [10]. Keeping in mind this minor concentration of PBZ we choose a lesser concentration for our study. Some previous literature also examined the developmental effects of PBZ at the concentration of 10 µg/L (0.01 ppm) and 100 µg/L (0.1 ppm) and found liver, intestinal and pancreatic cell defect [14,15]. Furthermore, Glyphosate and Roundup were noticed resulting locomotion and aversive behavioral alteration concentration range from 10 µg/L (0.01 ppm) to 500 µg/L (0.5 ppm) and suggested that behavior is a more sensitive indicator of toxicity than the lethality [16]. Therefore, we did not exceed our higher concentration from 1000 µg/L (1 ppm) even for adult zebrafish. The behavior has been used as a sensitive and non-invasive method to monitor environmental contamination because it provides a link between physiological and ecological processes, providing an ideal platform to study the biosafety of pesticides [3].

Zebrafish is a widespread model for research in pharmacological and other chemical compounds owing to their salient features and behavior phenotypes as well as well-matched concurrence with rodent model experiments. A comprehensive set of behavioral test panel is available for zebrafish. They are most frequently studied for analyzing the underlying mechanism of the pharmacological compounds that alter behavior with many other findings, which can be translated in compiling results and data in large amounts leading to find a cure that can be translated for mammals and human disorders. The acclimation ability of zebrafish to a new environment makes it a good model for behavior and neuroscience studies [17,18]. The revolutionary developments in instrument and software made zebrafish an excellent in vivo animal model to understand the neural control of behavioral patterns in response to feeding, anxiety, predator avoidance, circadian rhythm, and other diverse behaviors [12,17,18]. Additionally, its small body size (around 3 cm long) makes it easy to handle and grow in a laboratory. For this study, we used zebrafish that have long been used as an animal model for biomedical research, particularly in developmental, toxicological, behavioral, and genetic studies [17,18]. In addition, zebrafish lay 200–300 eggs in a single time, the transparency of the embryos at initial stages of zebrafish larvae provide an excellent opportunity for observing the interaction of injected particles with the internal organs.

Earlier studies reported that low dose PBZ has an acute toxic effect on antioxidant enzymes in fish [19] and this observation promote us to explore the biosafety of PBZ by conducting behavioral assessments and neurochemical alteration at larvae and adult stage. Furthermore, we proposed a possible mechanism for the behavioral alteration caused by PBZ exposure using different biomarkers from the brain tissue. Even though behavioral indicators offer insights into various levels of ecological and physiological aspects of toxicology, but the molecular markers could make the behavioral insight more vivid and clearer. Our findings showed that exposure to PBZ reduced the locomotor activity in the larvae and induced the anxiolytic behavior in adult zebrafish at the lowest concentrations for the first time.

## 2. Results

### 2.1. Overview of Experimental Design

**Figure 1:**
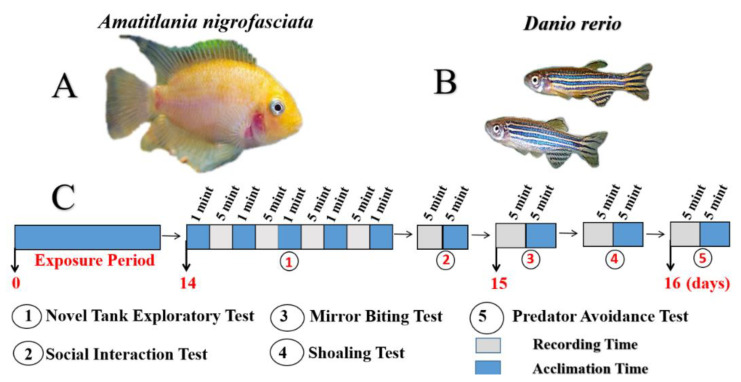
Experimental design for Paclobutrazol (PBZ) toxicity test in zebrafish by multiple behavior analysis. (**A**) The convict cichlid (*Amatitlania nifrofasciata*) was used as predator fish. (**B**) The AB strain zebrafish used in this study. (**C**) Five major behavior tests were performed after the adult zebrafish were exposed to PBZ at different concentrations (100 µg/L and 1000 µg/L) for fourteen-days. At 14 days post-experiment, novel tank, and social interaction assays were performed. At 15 days post-experiment, mirror biting and shoaling tests were performed. At 16 days post-experiment, predator avoidance test, and biochemical assay were performed.

In this study, we evaluated the locomotor activity in zebrafish larvae after exposure of 10 µg/L (0.01 ppm) and 100 µg/L (0.1 ppm) of PBZ as acute toxicity test. For chronic toxicity tests, we evaluated the behavioral changes in adult zebrafish after exposed to 100 µg/L (0.1 ppm) and 1000 µg/L (1 ppm) of PBZ. The PBZ dosage used in this study is closed to the environmental concentration been reported in groundwater and surface water of the river [10]. For the larvae test, zebrafish aged at 96 hpf (hour post-fertilization) were transferred to 48 well plates and then exposed to PBZ for 24 h. Their locomotor activities in terms of total distance traveled, rotation, and burst movement were measured at 120 hpf for comparison. For adult fish tests, zebrafish aged at around six months old were exposed to 100 µg/L (0.1 ppm) and 1000 µg/L (1 ppm) of PBZ for 14 days and their behavioral alterations in terms of the novel tank, mirror biting, predator avoidance, social interaction and shoaling tests and brain biomarker changes were measured at the end of the experiment (summarized in Figure 1). Finally, the brain tissues were dissected from control or PBZ-exposed zebrafish to perform ELISA for biomarker quantification and comparison.

### 2.2. Paclobutrazol Treatment Altered Locomotion Activity in Zebrafish Larvae

**Figure 2:**
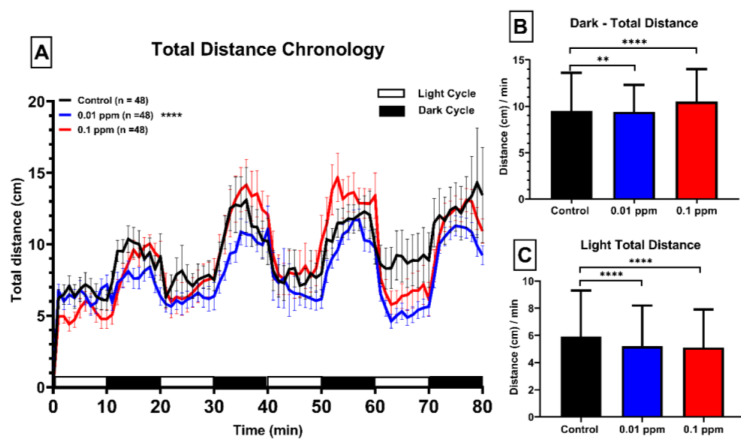
Total distance chronology of zebrafish larvae after exposure of Paclobutrazol. (**A**) The total distance traveled in either light or dark phases of zebrafish larvae after treated with 0 (black color), 10 µg/L (0.01 ppm) (blue color), or 100 µg/L (0.1 ppm) (red color) ppm of Paclobutrazol. Quantitative comparison of total distance traveled in the light phase (**B**) or dark phase (**C**) for zebrafish larvae after exposure of Paclobutrazol. The data are expressed as the means ± S.E.M. and analyzed by the one-way ANOVA with the Geisser–Greenhouse correction (** *p* < 0.01, **** *p* < 0.0001).

The locomotor activity is one of the major activity controlled by the brain and muscle, any impairment to the locomotion activity of the zebrafish directly indicate the side effects the tested chemicals have caused on to the model organism, in our case, the locomotor activity of larvae was measured by the considering three major endpoints like total distance traveled, burst movement and rotatory movement by each group. The total distance traveled in 10 µg/L (0.01 ppm) PBZ-treated group reduced in both dark and light periods. On the other hand, 100 µg/L (0.1 ppm) PBZ treated group of larvae covered the higher distance in the dark period and lower in the light period as compared to the control group (Figure 2B,C). When we combined and analyzed the dark and light cycle together, we did not find any significant difference between 100 µg/L (0.1 ppm) (red color) and control group (black color). However, the 10 µg/L (0.01 ppm) group (blue color) had a significant reduction of total distance covered in their combined dark and light cycle period (Figure 2A).

**Figure 3:**
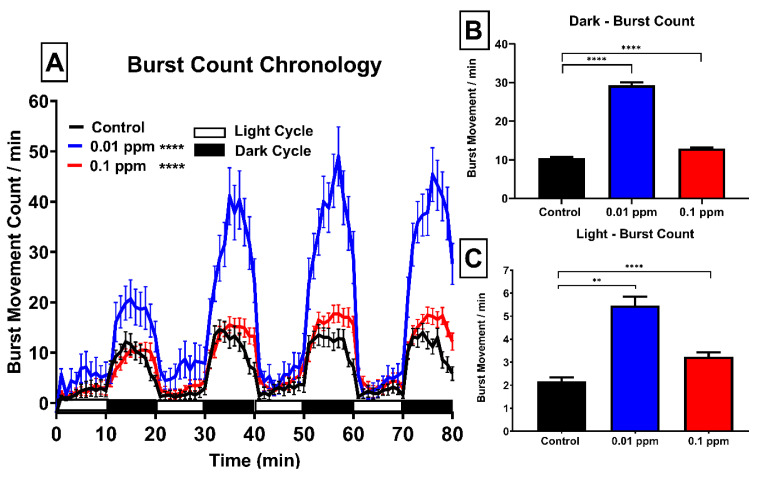
Total burst counts chronology of zebrafish larvae after exposure of Paclobutrazol. (**A**) Total burst movement in either light or dark phases of zebrafish larvae after treated with 0 (black color), 10 µg/L (0.01 ppm) (blue color) or 100 µg/L (0.1 ppm) (red color) ppm of Paclobutrazol. Quantitative comparison of burst movement in the light phase (**B**) or dark phase (**C**) for zebrafish larvae after exposure of Paclobutrazol. The data are expressed as the means ± S.E.M. and analyzed by the One-way ANOVA with the Geisser–Greenhouse correction (** *p* < 0.01, **** *p* < 0.0001).

Next, we measured the burst movement in zebrafish larvae. Burst movement is a spontaneous movement of zebrafish larvae in response to stimuli and can be used as an indicator to evaluate how larvae fish copy with environmental challenges. Generally, during positive phototaxis (testing arena is dark), a rate of burst initiations elevated. The previous study discovered that ventromedial serotonergic neurons played an important role in zebrafish larvae burst swimming [20]. In addition, these movements are in response to optomotor response (OMR) and associated with the prey orientation behavior. Based on these findings, in human behavior, this movement may be resemble their reflect movement when they sense danger or even a hyperactivity behavior that may indicate an anxiety condition [21,22]. In addition, the quantification of the burst movement offers a simple method to identify the functional reliability of the motor neuronal system [23]. Furthermore, this index has also been used to discover small neuroactive drugs molecules via high throughput behavioral assay in a previous study [24]. We counted the burst movement event per minute and observed higher burst movement events in both concentrations of PBZ treated groups compared to their control counterpart. After quantification, the burst movement count display significantly elevated in both 10 µg/L (0.01 ppm) and 100 µg/L (0.1 ppm) PBZ treated groups at both light and dark cycles comparing to the control group (Figure 3A–C). It is intriguing to find the relative counts for burst movement in 10 µg/L (0.01 ppm) PBZ treated group (highlighted by blue color) can reach around three-folds higher than the control. However, the burst count can only reach 1.5-folds higher than control when larvae exposed PBZ at 100 µg/L (0.1 ppm, highlighted by red color) dose. This result demonstrates that PBZ treatment can trigger burst movement in a non-linear manner (inverted U shape pattern), showing more superior elevation in low dose than a high dose of PBZ exposure in zebrafish larvae.

**Figure 4:**
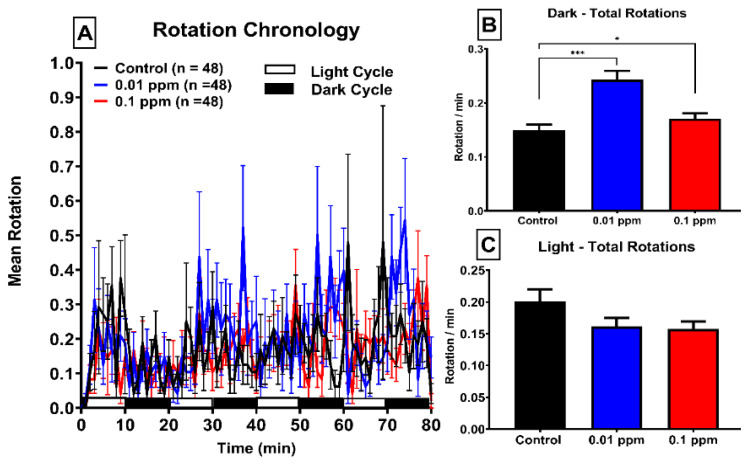
Rotation chronology of zebrafish larvae after exposure of Paclobutrazol. (**A**) Mean rotation in either light or dark phases of zebrafish larvae after treated with 0 (black color), 10 µg/L (0.01 ppm) (blue color) or 100 µg/L (0.1 ppm) (red color) ppm of Paclobutrazol. Quantitative comparison of rotation movement in the light phase (**B**) or dark phase (**C**) for zebrafish larvae after exposure of Paclobutrazol. The data are expressed as the means ± S.E.M. and analyzed by the one-way ANOVA with the Geisser–Greenhouse correction (* *p* < 0.05, *** *p* < 0.001).

Keeping in mind the circular shape of the 48-well plate used in locomotion test, fish rotation or its rotatory movement were also calculated. Rotation reflects motor aspects of zebrafish swimming and their movement orientations [25]. The rotation movement event is defined as the total rotation count that higher than 0.1 mm diameter and 60° of back angle per minute. According to our data, the mean rotation had no significant difference between PBZ-treated and control groups when four dark-light cycles were merged and compared (Figure 4A). However, when dark and light cycle data were separately estimated, we found a significantly high rotation movement during the dark period in both PBZ-treated groups (Figure 4B). The rotation movement during the light period in both PBZ-treated groups, on the contrary, displays no difference to their control counterpart (Figure 4C). This result demonstrates PBZ can trigger higher rotation movement (or moving direction changes) in the dark phase in zebrafish larvae. Similar to the burst count movement, PBZ treatment can trigger rotation movement in a non-linear manner (inverted U shape pattern), showing more superior elevation in low dose (0.01 ppm) than a high dose (0.1 ppm) of PBZ exposure in zebrafish larvae.

### 2.3. Zebrafish Display Anxiolytic Response in Novel Tank Test after Paclobutrazol Exposure

**Figure 5:**
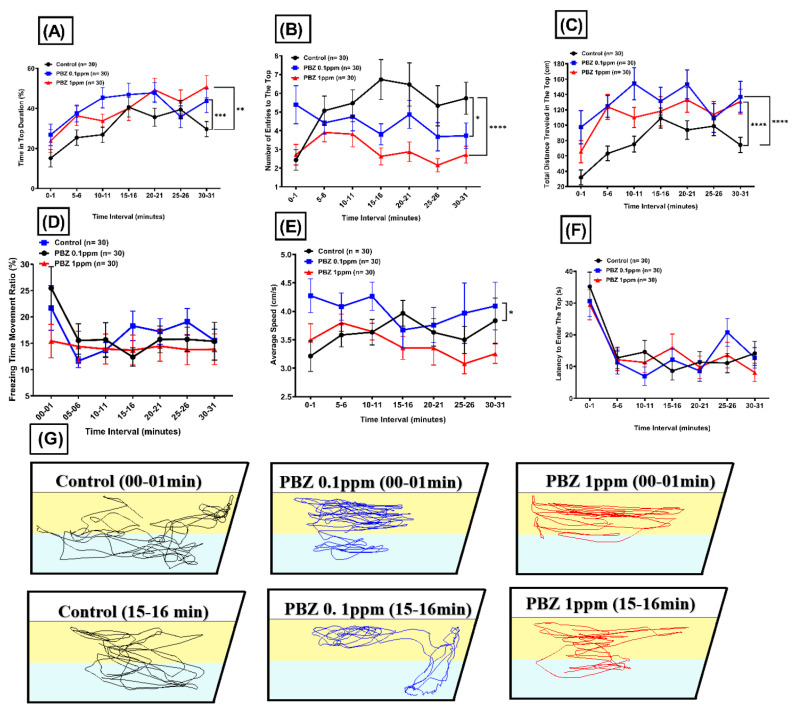
Novel tank exploratory behavior test for adult zebrafish after exposed to different doses of Paclobutrazol. The measured behavioral endpoints including: (**A**) time in top duration, (**B**) average speed, (**C**) freezing time, (**D**) number of entries in the top, (**E**) latency to enter in the top, and (**F**) total distance travel in the top. (**G**) Trajectories of adult zebrafish in novel tank assay after exposed to 0 (control), 100 µg/L (0.1 ppm), and 1000 µg/L (1 ppm) of Paclobutrazol (PBZ). The data are expressed as the means ± S.E.M. and were analyzed by the one-way ANOVA (* *p* < 0.05, ** *p* < 0.01, *** *p* < 0.001, **** *p* < 0.0001).

The exploratory behavior in the novel environment for adult zebrafish was analyzed after fish were continuously exposed to 100 µg/L (0.1 ppm) or 1000 µg/L (1 ppm) PBZ for 14 days. The fish were individually transferred into the novel tank, and their locomotor activity was recorded for the first minute immediately after animals were transferred to the testing tank. Then fish exploratory activity toward a novel environment was video recorded at 4–5, 9–10, 14–15, 19–20, 24–25, and 29–30 min for comparison (Figure 1C). The video was analyzed by idTracker software and six behavioral endpoints can be measured. The percentage of time spent in the top and total distance traveled at the top was higher in both treatment groups. However, the number of entries in the top marked lower (Figure 5A–C). The average speed of fish treated with 100 µg/L (0.1 ppm) PBZ became noticed as lower and we did get any significant difference for freezing and latency to enter the top (Figure 5D–F). By tracking fish moving trajectories, we clearly demonstrated that zebrafish display typical anxiolytic behavior which spent more time on the top of the tank after exposed to PBZ at both tested dose (Figure 5G).

### 2.4. Paclobutrazol Treatment Reduce Aggressiveness for Adult Zebrafish

**Figure 6:**
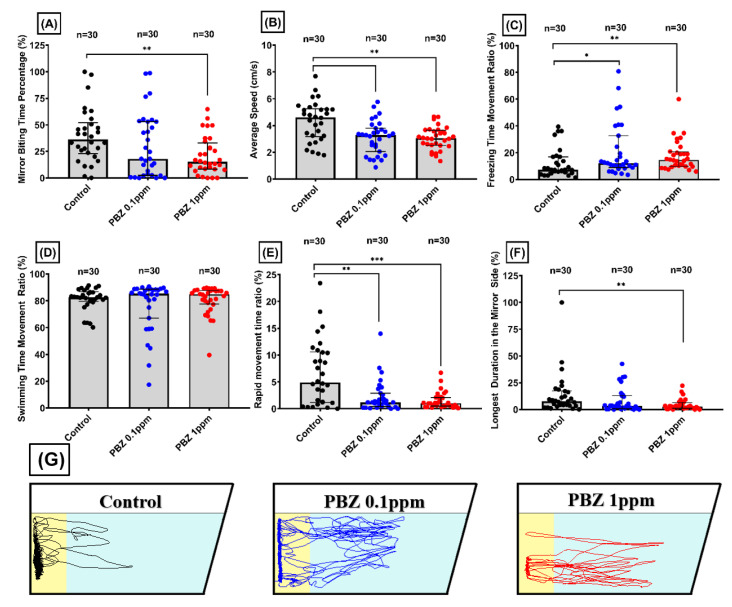
Aggressive behavior test for adult zebrafish after exposed to different doses of Paclobutrazol. Six behavioral endpoints were measured as: (**A**) mirror biting time, and (**B**) average speed of control and treatment group. (**C**) Freezing time ratio of all tested group, (**D**) swimming time movement ratio of all groups, (**D**) swimming time movement of all the group, (**E**) rapid movement ratio, and (**F**) duration of stay in the mirror side. (**G**) Trajectories of adult zebrafish in mirror biting assay after exposed to 0 (control), 100 µg/L (0.1 ppm), and 1000 µg/L (1 ppm) of Paclobutrazol (PBZ). The data are expressed as the means ± S.E.M. and were analyzed by the one-way ANOVA (* *p* < 0.05, ** *p* < 0.01, *** *p* < 0.001).

Mirror biting assay is an efficient and simple method to analyze the aggressive behavior of fish. Aggressive behavior was evaluated by placing a mirror to the back sidewall of the tank and counting how the high frequency of the tested fish interact with their mirror images. After PBZ exposure, zebrafish were transferred to the assay instrument, and videos were recorded for five minutes after one minute of acclimation. The entry of the fish most towards the mirror would be considered an opponent and most away from the mirror as avoidance. Less mirror biting interesting was observed in fish exposed to 1ppm PBZ showing marked lower mirror biting percentage (Figure 6A) and duration of stay in the mirror side (Figure 6F). Our data showed that both PBZ treated groups had lower rapid movement (Figure 6E), average speed (Figure 6B), and higher freezing time percentage (Figure 6C). We did not find any significant difference between the control and PBZ-treated groups concerning swimming time (Figure 6D). The projected moving trajectories showed that PBZ-treated fish indeed display less mirror visiting behavior (Figure 6G).

### 2.5. Paclobutrazol Treatment Had No Effect on Predator Avoidance Behavior

**Figure 7:**
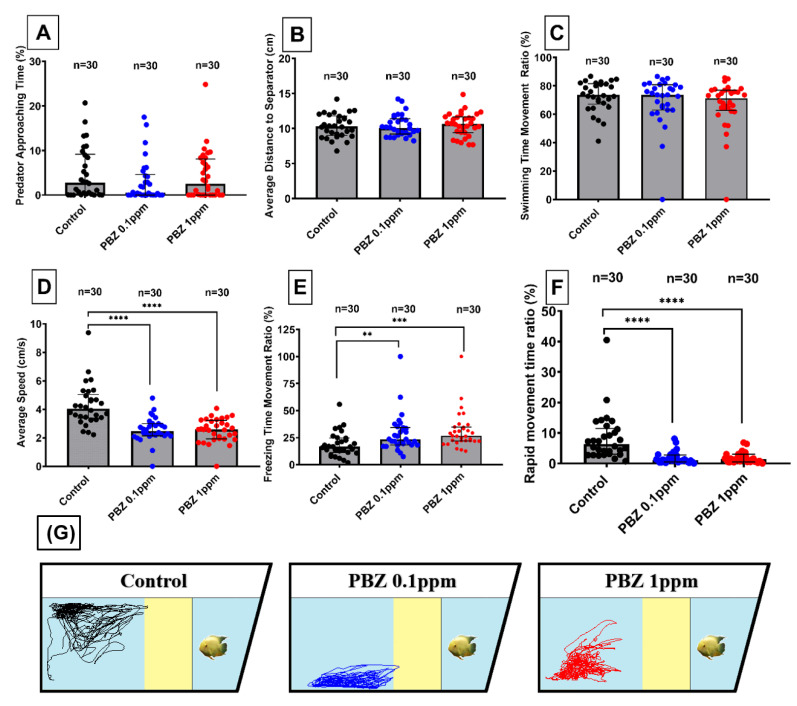
Predator avoidance test for adult zebrafish after exposed to different doses of Paclobutrazol. Six endpoints were measured including: (**A**) predator approaching time, (**B**) average distance to the separator, (**C**) swimming time movement, (**D**) average speed, (**E**) freezing time movement, and (**F**) rapid movement time ratio. (**G**) Trajectories of adult zebrafish in predator avoidance assay after exposed to 0 (control), 100 µg/L (0.1 ppm), and 1000 µg/L (1 ppm) of Paclobutrazol (PBZ). The data are expressed as the means ± S.E.M. and were analyzed by the one-way ANOVA (** *p* < 0.01, *** *p* < 0.001, **** *p* < 0.0001).

The predator avoidance is an innate behavior of zebrafish when it engages its predator. To test whether chronic exposure to PBZ can alter such innate behavior, we placed the predator fish convict cichlid *(Amatitlania nigrofasciata)* into a tank separated into two parts by using a transparent separator [26]. The test zebrafish swum away from the separator was considered as predator avoidance. Different independent endpoints such as predator approaching time, average distance to the separator, swimming time movement were calculated using idTracker software. PBZ treated groups at either 100 µg/L (0.1 ppm) or 1000 µg/L (1 ppm) displayed typical predator avoidance behavior at the same level as their control group. By statistic comparison, the predator approaching time and average distance to the separator were not showed a significant difference (Figure 7A,B). Conversely, the average speed (Figure 7D), rapid movement (Figure 7F), and freezing time movement (Figure 7E) decreased in PBZ treated groups as compared to the control. The ratio of swimming time movement had no significant difference in both treatment groups (Figure 7C). Furthermore, projected moving trajectories also showed that both PBZ-treated groups remain away from the predator as did by the control (Figure 7G).

### 2.6. Paclobutrazol Had No Alteration Effect on Social Interaction Behavior

**Figure 8:**
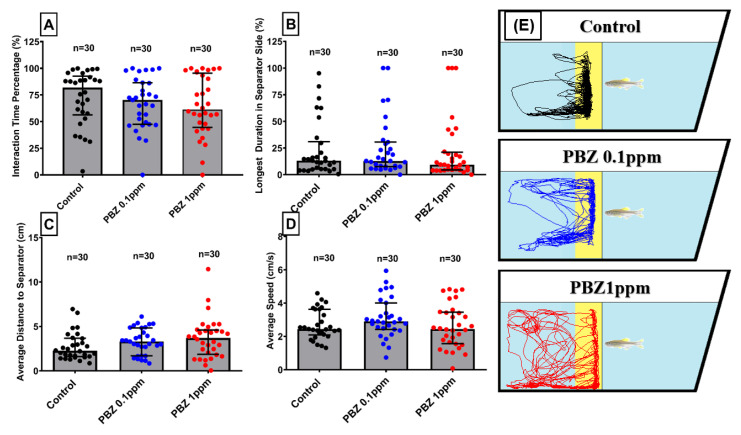
Social interaction test for adult zebrafish after exposed to different doses of Paclobutrazol. Four endpoints were measured including: (**A**) interaction time percentage, (**B**) duration of stay in separator side, (**C**) average distance to the separator, and (**D**) average Speed. (**E**) Trajectories of adult zebrafish in social interaction assay after exposed to 0 (control), 100 µg/L (0. 1 ppm), and 1000 µg/L (1 ppm) of Paclobutrazol (PBZ). The data are expressed as the means ± S.E.M. and were analyzed by the one-way ANOVA.

Zebrafish has been reported to display active social interaction and shoaling behavior [27], thus provide us an ideal model to test whether chronic PBZ treatment at 100 µg/L (0.1 ppm) to 1000 µg/L (1 ppm) levels can alter social interaction and shoaling behaviors. Interaction time percentage, longest duration in separator side, the average distance to the separator, and average speed were measured to analyze their conspecific interaction behavior. For conspecific social interaction assay, our result showed there was no significant difference in PBZ-treated groups as compared to control (Figure 8A–D). Locomotor trajectories for different PBZ-treated groups also showed similar interaction level with their control counterparts (Figure 8E).

**Figure 9:**
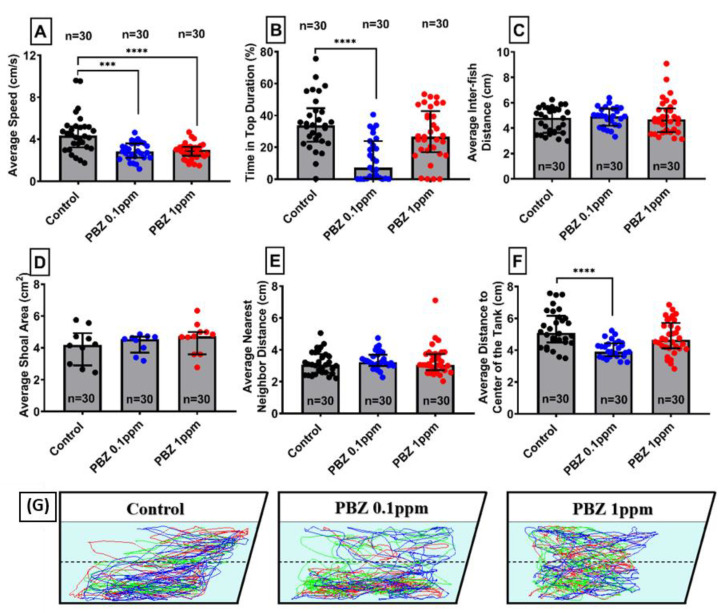
Shoaling test for adult zebrafish after exposed to different doses of Paclobutrazol. Six behavioral endpoints were tested including: (**A**) average speed, (**B**) time in top, (**C**) distance between individual fish, (**D**) average shoal area, (**E**) average nearest neighbor distance, and (**F**) average farthest neighbor distance. (**G**) Trajectories of adult zebrafish in shoaling assay after exposed to 0 (control), 100 µg/L (0.1 ppm), and 1000 µg/L (1 ppm) of Paclobutrazol (PBZ). The shoal size for every group was three fish. The data are expressed as the means ± S.E.M. and were analyzed by the one-way ANOVA (*** *p* < 0.001, **** *p* < 0.0001).

Shoaling is an innate behavior of fish, swim together in order to reduce anxiety and the risk being captured by the predators. Zebrafish is a fish species live in a group and swim together to minimize the anxiety upon the engagement of predator [28]. This innate behavior of zebrafish also helps to improve predator avoidance as well as food chasing efficiency. After PBZ exposure, although the low dose PBZ-treated fish swum slower (Figure 9A) and spent more time in the bottom (Figure 9B), all other endpoints like average inter-fish distance (Figure 9C), average shoal area (Figure 9D), and average farthest and nearest distance (Figure 9E,F) had no significant difference. Locomotor trajectories for different PBZ-treated groups also showed a similar shoaling patterns with their control counterparts (Figure 9G).

### 2.7. Paclobutrazol Exposure Downregulate Neurotransmitters

**Figure 10:**
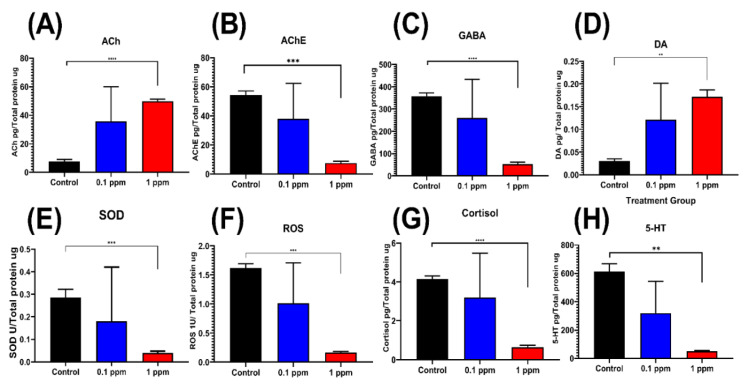
Biochemical Assay for neurotransmitters and enzymes content in zebrafish brains after exposed to different doses (0.1 and 1 ppm) of Paclobutrazol. Eight biomarkers were tested including (**A**) acetylcholine (ACh), (**B**) acetylcholine esterase (AChE), (**C**) γ-Aminobutyric acid (GABA), (**D**) dopamine, (**E**) superoxide dismutase (SOD_, and (**F**) reactive oxygen species (ROS), (**G**) cortisol and (**H**) serotonin (5-HT). All data are expressed as means ± S.E.M and significance were measured and analyzed by one-way ANOVA (** *p* < 0.01, *** *p* < 0.001, **** *p* < 0.0001).

Based on larvae and adult fish tests, our behavioral assay clearly demonstrated PBZ indeed can induce behavioral alteration in both larvae and adult fish even when the exposed dose is relatively low. Why PBZ exposure can trigger such behavioral abnormality in zebrafish is an intriguing question for us. To probe this question, we attempted to evaluate the possible underlying mechanism by performing biochemical assay targeting some crucial biomarkers in the brain. Hyperactive in larvae, reduced innate aggressive and anxiolytic behavior can be correlated with the neurotransmitters and some oxidative stress enzymes in the brain. By ELISA (enzyme-linked immunosorbent assay) measurement, our data showed acetylcholine (Figure 10A) and dopamine (Figure 10C) contents displayed significant elevation in high dose (1 ppm) PBZ treated zebrafish. The other biomarkers like acetylcholine esterase (AChE, Figure 10B), γ-Aminobutyric acid (GABA, Figure 10C), SOD (Figure 10E), reactive oxygen species (ROS, Figure 10F), cortisol (Figure 10G) and serotonin (5-HT, Figure 10H) showing decreased expression after PBZ treatment. Data of the present study revealed that zebrafish challenged with 1 ppm PBZ significantly decrease brain SOD level which is a critical enzyme in the suppression of oxidative stress (Figure 10E). The low cortisol in the brain in PBZ-treated fish suggest fish is less stress and supporting the anxiolytic phenotype detected in the novel tank assay. In addition, the low GABA level in the brains for PBZ-treated fish (Figure 10C) might be associated with the slow swimming speed detected in mirror biting, predator avoidance, and shoaling assays.

## 3. Discussion

To improve the productivity of the crops, enhancing the morphological, chemical, and physical features for economic benefits, pesticides are used at a very large scale by cultivators/farmers. PBZ is one such farming additive of triazole family used as a growth inhibitor, an inducer of morphological modification of leaves, and increase root density in plants. PBZ specifically inhibits the ent-kaurene oxidase enzyme activity to block gibberellins biosynthesis in plants [29]. Gibberellin a diterpenoid phytohormone helps stimulate cell elongation and growth enhancement amongst other activities in the plants. Other than inhibiting gibberellins, PBZ have a wide variety of functions making it one of the widely used pesticide in the world [9,30]. It was first registered by United States Environmental Protection Agency (U.S. EPA) in 1985 and started to be exploited randomly besides learning about the toxicological, neurological, and biological side effects. The biological toxicity of PBZ is still very less explored and hence needed to be studied at various physiological, behavioral, biochemical, and molecular levels to understand the sustainability and toxicity criteria of PBZ.

PBZ is a very stable compound and its high mobility provides its potential for bioaccumulation in the ecological system. Being a very stable compound, the mobility of PBZ is a serious concern in soil and underground water [11,14]. Therefore, it is necessary to analyze and assess the toxicity criteria, to understand the negative impacts it can cause to the elements of the ecological system. In this aspect considering water bodies as a point of contact with irrigation and cultivation, it is necessary to assess the toxicity in aquatic organisms. Recently, the potential toxicity of PBZ has been proposed in diverse vertebrates like zebrafish and rats. De Castro et al. reported alterations in locomotion and acoustic startle reflex for rat pups when their mother exposed to PBZ in the drinking water [31]. Yekti et al. reported zebrafish embryos exposed to PBZ can disrupt the heart and craniofacial cartilage development and decreases their survival and hatching rates [32]. Wang et al. reported zebrafish embryos exposed to PBZ can disrupt the digestive system and retina development [14,15]. Ghane et al. reported tilapia fingerlings exposed to sub-lethal PBZ can alter physiological conditions and induce stress [33].

Compared to embryonic toxicity, the potential toxicity of PBZ is seldom be addressed at the adult stage. Although some toxicity studies have been performed on adult zebrafish from triazole family, indicating, altered behavioral responses such as hypoactivity, prominently at 100 µM but variable at lower doses; concluding that relatively higher doses are required to induce deformities and impair locomotion activities and oxidative respiration in early developmental stages of zebrafish [34]. The locomotion activities are the most conspicuous and leading indicators of behavior analysis of formulated chemical, pharmacological compounds, and drugs. As the locomotor activity directly gets affected by the central nervous system (CNS) it gives a clear idea about any internal brain function impairment [35].

The important outputs of our results are we provided solid evidence at both behavioral and biochemical levels showing the exposure of environment relevant level of PBZ indeed can induce behavior alterations at both larvae and adult fish for the first time. The zebrafish larvae showed hyperactivity as treated with the lowest dose of 10 µg/L and 100 µg/L of PBZ (0.01, 0.1 ppm). Furthermore, the higher burst movement and rotation suggested that PBZ treatment alter the swimming pattern. The chronic exposure to PBZ in adult zebrafish can induce anxiolytic behavioral effects as it spent more time and higher traveled distance on the top of the tank, and the similar nicotine-related anxiolytic effect was also reported by Levin et al. [36]. Based on cortisol measurement, we suggest this anxiolytic behavior in PBZ-treated fish is associated with the reduced level of cortisol in their brain. In addition, the reduced level of GABA might be associated with the reduced swimming speed of PBZ-treated fish in several assays like mirror biting, predator avoidance as well as shoaling assays.

Our findings revealed that zebrafish chronically treated with PBZ at 1 ppm level displayed significant aggression reductions. Fontana et al. reported that aggressive behavior is coupled with positive allosteric modulators of GABA_A_ receptors [37], so the possible mechanism for this is the downregulation of GABA. However, more studies have to confirm mechanism for aggressive behavioral alteration discovered yet because aggression is a complex behavior that influences social relationships and can be seen as adaptive or maladaptive depending on the context and intensity of expression. The present data also showed that the inhibitory effect of PBZ on AChE, cortisol, serotonin, SOD, and GABA. However, ACh and dopamine levels in the brain were elevated after PBZ exposure. The downregulation of biomarkers related to anxiety and stress and elevation of the ACh and dopamine suggested that PBZ had an anxiolytic effect and the brain may be the target area for the early anxiolytic effect of PBZ mediated by cholinergic transmission as reported by Noor et al. [38].

**Figure 11:**
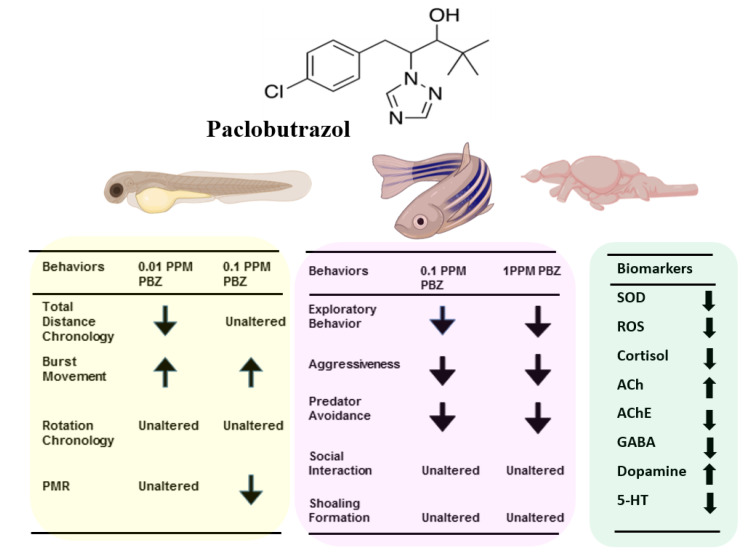
Schematic summary of the behavioral and biochemical alterations in larvae and adult zebrafish after exposed to different doses of Paclobutrazol. The left yellow panel summarizes the behavioral alterations in the larvae stage, middle pink panel summarizes the behavioral alterations in the adult stage, and right green panel summaries the biomarker content in adult brains after exposed to Paclobutrazol.

In the light of this study, we can confirm our hypothesis that PBZ exposure induces anxiolytic behavioral changes as both treated group to have higher time in top duration, lower number of entries in the top explaining fish remain on the top, which is robust in higher concentration. Finally, the total distance in the top is higher in both treatment groups explaining they mostly swim on the top also observed aggressive response deficit. If aggressiveness deficit it results in serious problem of resource utilization like nesting and foraging site and mate disputes. Furthermore, the current toxicity levels of PBZ in water bodies responsible to decrease the exploratory behavior, lead the fish to unbalance in its habitats (all behavioral and biochemical alterations were summarized in Figure 11). All those behavioral alterations might generate ecotoxicity and sharply reduce their survival fitness for chronic exposure to PBZ. In this consideration, more behavioral tests like circadian rhythm, mating tests, trans-generation transmission, and whole life span monitoring are considered necessary to elucidate more chronic toxicity induced by PBZ in a model animal system.

## 4. Experimental Methodology

### 4.1. Ethics Statement and Animal Breeding

The protocol of this study has been approved by Institutional Animal Care and Use Committee (IACUC) at Chung Yuan Christian University (Approval No. 109001, issue date 15 January 2020) for proper use of zebrafish. All studies were carried out in strict accordance with the guidelines of the IACUC. One female and two males were transferred into a spawning tray and separated them by placing a transparent separator between them. After 24 h, the separator was removed and embryos were collected, sanitized, and placed at 28 °C after a few hours. All the dead embryos were removed every day until day three.

### 4.2. Larvae and Adult Exposure to Paclobutrazol

Zebrafish AB strain stock was housed in a system connected with a recirculating aquatic system at 26.5 °C with a 10/14 dark-light cycle and fed two times a day with artemia according to the breeders in an Association for Assessment and Accreditation of Laboratory Animal Care (AAALAC) approved animal facility. Zebrafish embryos aged at 4 dpf (days post-fertilization) were placed into a petri dish and exposed to different concentration of 10 µg/L and 100 µg/L of PBZ (purchased from Sigma-Aldrich, cat. Number 46046). Wild-type adult zebrafish aged at 4–6 months with mixed genders were incubated in 10L containers with either 100 µg/L (0.1 ppm) or 1000 µg/L (1 ppm) PBZ. During the 14 days incubation with PBZ, adult fish were routinely fed and the water of the whole tank was changed every after two days to maintain the constant concentration of PBZ exposure.

### 4.3. Exploratory Behavior of Larvae

The zebrafish larvae were shifted to clear 48-well plates and put 800 µL of fish water in each well. After 30 min of acclimation time, the locomotor activity of zebrafish larvae aged at 120 hpf was recorded for an hour and twenty minutes using 10-min alternative light (visible light; 75% intensity) and dark cycle (infrared light). The infrared light remained on throughout the recording session. However, the visible light was switched on after every 10 min for the light cycle, and then switched off for the dark period. Later we did the behavioral assay such as total distance covered, turning frequency, and bursts during every 10 mints of the dark and light cycle using the ZebraBox instrument equipped with infrared camera and their tracking extension software system according to a previous protocol [39,40,41]. A total of 80 min of recording with swimming data were collected. Swim speed thresholds were set based on above described studies to define three different speed thresholds. These speeds included bursting (>2.0 cm/s), which were short, intermittent, and powerful bouts of activity, cruising (0.5 > s < 2.0 cm/s), covering most of the commonly measured larval speeds, and freezing (<0.5 cm/s) during which larvae displayed minimal or non-moving activity. Later, the video was analyzed further to obtain the rotation counts by Zebralab software. Rotation count was defined as one event if the rotation was higher than the minimum diameter (0.1 mm) and back angle (60°) within one minute. In all behavioral assay videos, the background is subtracted detection sensitivity was set 20. The detection sensitivity value is actually the greyscale pixel intensity value, and any pixel in the video darker than this threshold was detected as an animal.

### 4.4. Adult Behavior Analysis

After 14 days of incubation, the behavior assay was immediately done according to the protocol listed in Figure 1. To evaluate the behavior of adult fish, we used Zebrafish tower, which is a high-throughput screening tool for zebrafish behavioral studies reported in our previous publication [18]. The locomotor activity (time in the top, time in bottom, average speed, and distance between individual fish) and complex behavior (shoaling, aggressiveness, social interaction, and predator avoidance) of each individual fish were recorded and digitized followed by quantitative analysis by using open-access software of idTracker [42] (experimental workflow was summarized in Figure 1).

### 4.5. Tissue Preparation and Total Protein Estimation

After performing all behavioral assay, the adult fish were sacrificed with a high concentration of tricaine (MS-222) solution (200 mg/L). For each biochemical assay, the fish brain tissues were collected and combine three brain tissues of zebrafish to prepare an independent homogenate. Phosphate buffer saline (PBS) at pH 7.2 was added to each sample then centrifuged at 15,000 rpm at 4 °C for 15 min. Supernatant was separated and store at −80 °C for further analysis. A BCA Protein Assay Kit (23225, Thermo Fisher Scientific, Waltham, MA, USA) was used to determine the concentration of total protein. We used a microplate reader (Multiskan GO, Thermo Fisher Scientific, Waltham, MA, USA) at 562 nm to analyze the color formation during BCA.

### 4.6. Determination of Neurotransmitters

For measuring neurotransmitter content in the brain tissues, brains from three fish were pooled into one sample and four duplicates per group were performed to ensure consistency. The relative contents ACh (acetylcholine), AChE (acetylcholine esterase), GABA (γ-aminobutyric acid), dopamine, serotonin, and cortisol were measured using sandwich-based ELISA (enzyme-linked immunosorbent assay) kits (ZGB-1585, ZGB-1637, ZGB-1574, ZGB-1573, ZGB-1572, Zgenebio, Taipei, Taiwan) following protocol they mentioned. Shortly, 45 µL of dilution buffer and 5 µL of brain homogenate was placed on to the specific antibody-coated ELISA plate. Then, 100 µL of the working reagent solution mix was added into each well. After 15 min of incubation, the absorbance was analyzed at 450 nm using a microplate reader (Multiskan GO, Thermo Fisher Scientific, Waltham, MA, USA).

### 4.7. Analysis of Reactive Oxygen Species and Antioxidant Capacity

The reactive oxygen species (ROS) were analyzed using the ELISA kit (ZGB-1561, Zgenebio, Taipei, Taiwan) by following the protocol provided by the company. The absorbance values were determined at 450 nm. The production of superoxide dismutase (SOD), the strong antioxidant enzyme in the brain was determined using the ELISA kit (ZGB-1604, Zgenebio, Taipei, Taiwan). The color formation was analyzed by a microplate reader at a wavelength of 450 nm.

### 4.8. Statistical Analysis

The statistical analysis and data visualization were carried out with GraphPad Prism (GraphPad Software, Inc., version 7.00, La Jolla, CA, USA). The comparison was made using one-way ANOVA, parametric, or non-parametric test, depending on data normality for significance determination. The data shown are presented with mean ± SEM with *p* < 0.05 regarded as statistically significant.

## 5. Conclusions

To sum up: this is the first study to demonstrate the behavioral alterations in both larvae and adult zebrafish after chronic exposure of relatively low concentration of PBZ by conducting a battery of zebrafish behavioral assays. In the zebrafish larvae, the alterations were shown in their hyper-locomotor activity and reduced photomotor response. Meanwhile, loss of aggressiveness and predator avoidance behavior, followed by anxiolytic exploratory behavior were exhibited by the adult zebrafish after chronically treated with PBZ. In addition, several abnormal levels in neurotransmitters or stress hormones were also displayed by the PBZ treated-fish, which might elucidate their altered behaviors (summarized in Figure 11). Taken together, since PBZ is a widely-used plant growth regulator, we suggest that it is necessary to conduct more thorough tests for its biosafety and bioaccumulation.
